# Concentric remodeling and the metabolic-associated steatotic liver disease in patients with type 1 diabetes: an exploratory study

**DOI:** 10.1007/s00592-024-02365-3

**Published:** 2024-09-17

**Authors:** Klaudia Czarnik, Zbigniew Sablik, Anna Borkowska, Jarosław Drożdż, Katarzyna Cypryk

**Affiliations:** 1 Department of Internal Diseases and Diabetology, Medical University of Lodz, Central Teaching Hospital in Lodz, 251 Pomorska Street, 92-213 Lodz, Poland; 2 Department of Cardiology, Medical University of Lodz, Lodz, Poland; 3 Department of Digestive Tract Diseases, Faculty of Medicine, Medical University of Lodz, Lodz, Poland

**Keywords:** Diabetes type 1, Diabetes complications, Left ventricle remodeling, Metabolic-associated steatotic liver disease

## Abstract

**Introduction:**

Diabetic cardiomyopathy in young patients with type 1 diabetes (T1D) usually presents as asymptomatic diastolic heart dysfunction with left ventricle (LV) remodeling. Its prevalence seems to be underestimated. One of the factors seemingly influencing LV remodeling is a metabolic-associated steatotic liver disease (MASLD), which was extensively investigated in patients with type 2 diabetes but not with T1D. This study aimed to describe the correlation between MASLD risk and relative wall thickness (RWT) in young patients with T1D without heart failure symptoms or treatment.

**Materials and methods:**

Study participants were recruited at the inpatient diabetology department, in admission order. Patients underwent a set of laboratory tests and echocardiographic examinations. The risk of MASLD was estimated using fatty liver index (FLI). Acquired data was then statistically analyzed.

**Results:**

The study group consisted of 55 patients. 25 participants had RWT > 0.42, suggesting LV remodeling. Study participants did not differ in HbA1c, NT-proBNP, HDL, LDL, non-HDL, and uric acid concentrations. However, patients with RWT > 0.42 had higher FLI (40.97 vs. 13.82, *p* < 0.01) and BMI (27.3 vs. 22.5, *p* < 0.01) and differed in transaminase concentrations. Moreover, patients with RWT > 0.42 had significantly higher LV mass index (85.6 vs. 68.2 g/m2) and altered mitral ring velocities. In univariable logistic regression, FLI correlated with LV remodeling risk (OR 1.028, *p* = 0.05). The optimal cutoff point for FLI predicting the RWT > 0.42 was 26.38 (OR 10.6, *p* = 0.04, sensitivity 0.857, specificity 0.657).

**Conclusions:**

FLI correlates with RWT in patients with T1D independently of diabetes metabolic control and hypothetically may support recognizing T1D patients with a higher risk of LV remodeling.

## Introduction

Diabetic cardiomyopathy is a form of heart disease characterized by specific changes to cardiac structure and impaired cardiac function in patients with diabetes [1, 2]. Its pathogenesis is not fully understood as it seems to develop in patients without traditionally recognized risk factors, such as hypertension or dyslipidemia [1–3]. Features of diabetic cardiomyopathy may be identified in animal models and human studies early in the course of diabetes, usually many years before the heart failure (HF) symptoms appear [1–3].

In patients with type 1 diabetes (T1D), cardiovascular diseases (CVD) are the predominant cause of mortality and morbidity, and the prevalence of cardiac dysfunction is seemingly underestimated and somewhat underdiagnosed [1, 3, 4]. As recent study showed, patients with T1D develop symptomatic HF earlier by 10–23 years compared to patients without diabetes [3]. However, data on the prevalence of asymptomatic HF in T1D remains conflicting [1, 5–7].

Diabetic cardiomyopathy in young patients with T1D most commonly presents as asymptomatic diastolic heart dysfunction with left ventricle (LV) remodeling [8, 9].

LV remodeling, first defined in the 1990s, describes patterns of left ventricular structural responses to numerous factors including changing workload or cardiac injury [9, 10]. Remodeling models, such as concentric hypertrophy, eccentric hypertrophy, and concentric remodeling may be assessed using relative wall thickness (RWT) and LV mass index obtained using echocardiography [11, 12]. Multiple studies provide information on the correlation of LV remodeling with adverse cardiovascular outcomes [11, 13]. While LV remodeling has been extensively investigated in type 2 diabetes and seems to interconnect with metabolic syndrome, the data on it among T1D patients is scarce [14, 15].

One of the factors seemingly integrating various aspects of metabolic syndrome and influencing CV risk is the metabolic-associated steatotic liver disease (MASLD), previously named nonalcoholic fatty liver disease (NAFLD), which may correlate with increased atherosclerosis rate and insulin resistance and facilitate the deleterious effects of insulin deficiency and hyperglycemia, that accompany T1D [16, 17].

Given that, this study aimed to explore and describe the impact of MASLD on RWT in young patients with T1D naïve of HF symptoms or treatment.

## Materials and methods

The study was approved by the Bioethics Committee of the Medical University of Lodz (RNN/205/21/KE, July 13, 2021) and was performed according to the Declaration of Helsinki.

Study participants were recruited from October 1, 2021, through September 1, 2022, at the inpatient diabetology department in admission order. The only inclusion criterion was the diabetes type 1 diagnosis. Exclusion criteria were: other types of diabetes, metformin use, pregnancy, the presence of HF symptoms without the formal diagnosis of HF or treatment of HF, active hepatitis, alcohol abuse, and either alanine aminotransferase (ALT) or aspartate aminotransferase (AST) concentrations of at least two times above the upper reference limit. Study participants were not financially compensated for their participation.

All of the study participants received an information brochure and were given an opportunity to ask questions before signing a written informed consent. Participants’ medical history including drugs use, anthropometric measurements, physical examination, laboratory tests, and echocardiograms were included in the analysis.

## Biochemistry

Blood and urine samples were collected at 7:00 AM after a minimum eight-hour fast. Performed laboratory tests included (the abbreviation and the analysis method in brackets): the glycated hemoglobin (HbA1c, the immunoturbidimetric assay, DxC, Beckman Coulter), the albumin/creatinine ratio (ACR, the immunoturbidimetric and isotope dilution mass spectrometry, AU, Beckman Coulter), the N-terminal prohormone of brain natriuretic peptide (NT-proBNP, the chemiluminescent immunoassay method, Cobas, Roche), C-reactive protein (CRP, the immunoturbidimetric assay, DxC, Beckman Coulter), thyroid-stimulating hormone (TSH, the chemiluminescent immunoassay method, Alinity, Abbott), and aspartate aminotransferase (AST, the spectrophotometric method, DxC AU, Beckam Coulter), alanine aminotransferase (ALT, the spectrophotometric method, DxC AU, Beckam Coulter), gamma-glutamyltransferase (GTP, the spectrophotometric method, DxC AU, Beckam Coulter), creatinine (the spectrophotometric method, DxC AU, Beckam Coulter), uric acid (the spectrophotometric method, DxC AU, Beckam Coulter) and lipids (the spectrophotometric method, DxC AU, Beckam Coulter) concentrations.

## Echocardiography

All echocardiographic examinations were carried out by the same blind to patients’ characteristics researcher (ZS) according to the European Association of Cardiovascular Imaging recommendations using a commercially available ultrasound machine (GE Vivid Q, General Electric)[18]. Recorded images were digitally stored.

Either a 2-dimensional imaging technique or a 2-dimensional-directed M-mode was used to obtain linear measurements. The left ventricle mass was derived from the cube formula. The ejection fraction (EF) was calculated using the biplane method of disk summation. Mitral E and A velocities were obtained in an apical four-chamber view using pulsed-wave Doppler. Lateral and septal mitral annulus early diastolic (e’), peak systolic (s’), and peak late diastolic (a’) velocities were determined using Tissue Doppler in an apical four-chamber view. Mitral E/A and mitral E/e’ ratios were calculated. Isovolumic relaxation time, IVRT, and deceleration time of mitral E velocity, DT of mitral E velocity, were recorded in apical four-chamber view with pulsed-wave Doppler. The tricuspid plane systolic excursion, TAPSE, was acquired in an apical view using M-Mode tracing [19, 20].

## Relative wall thickness

The relative wall thickness was calculated as RWT = 2 x (LV diastolic posterior wall thickness/left ventricular end-diastolic dimension) [21]. The cut-off point for concentric remodeling was set at > 0.42 [21].

## MASLD risk

MASLD risk was assessed using the fatty liver index (FLI), considered a valid surrogate marker of hepatic steatosis, calculated as FLI = (e^0.953 x ln(TG concentration in mmol/L) + 0.139 x BMI + 0.718 + ln(GGT concentration in U/L) + 0.053 x waist circumference in cm—15.745^)/(1 + e^0.953 x ln(TG concentration in mmol/L) + 0.139 x BMI + 0.718 + ln(GGT concentration in U/L) + 0.053 x waist circumference in cm—15.745^) × 100[22].

## Statistical analysis

Statistical analysis was performed using the Statistica 13.1 software (StatSoft Inc., Tulsa, Oklahoma, United States). The Shapiro–Wilk test was used to assess the normality of the distribution of the study variables. Continuous variables are presented as mean and standard deviation (SD) or median and interquartile interval (25th and 75th percentile) according to their distribution. To compare groups with normally distributed data, a Student’s *t*-test was used. Mann–Whitney *U*-test was used to compare groups with non-normally distributed data. *P* < 0.05 was considered statistically significant.

Receiver operating characteristic curve (ROC) analysis was used to establish an optimal cut-off point for FLI predicting RWT > 0.42 occurrence. Then we explored potential cut-off points using the Chi2 test.

*Then we analyzed the interactions between variables statistically differing between RWT* ≤ *0.42 and RWT* > *0.42 groups and built univariate and multivariate regression models. P* values of less than 0.05 were considered statistically significant.

## Results

Out of sixty-five patients participating in the study, ten were excluded from the analysis due to missing data. The clinical characteristics of the study group are summarized in Table 1. In general, study participants had a mean age of 38.0 years (SD 9.6 years), their median BMI was 23.39 kg/m2 (IQR 21.60–27.30 kg/m^2^), and their median HbA1c was 8.05% (IQR 7.15–9.90%). The median RWT in the study group was 0.37 (IQR 0.34–0.40), the mean left atrium volume index (LA Vol index) was 24.1 ml/m^2^ (SD 4.8 ml/m^2^), and the mean left ventricle mass index (LV mass index) was 71.1 g/m^2^ (SD 13.0 g/m^2^). As there was only one study participant with an LV mass index exceeding the upper reference limit, we decided to recognize the pattern of concentric remodeling in the group and omit this parameter in further analysis.

**Table 1:** The clinical characteristics of the study group

Parameter	Mean (SD)	Median (IQR)
*Baseline characteristics*		
Age, years	38.0 (9.6)	
BMI, kg/m2		23.39 (21.61—27.25)
body weight, kg		67.0 (60.0—79.0)
DDI, U		40 (32—56)
DDI per body weight kg, U/kg		0.62 (0.46—0.73)
diabetes duration, years	21.8 (11.3)	
height, cm	168.9 (9.9)	
waist circumference, cm		80 (76—90)
*Biochemistry*		
ACR, mg/g		4.45 (2.55—12.55)
ALT, U/l		18.10 (13.25—27.00)
AST, U/l		20.95 (17.05—26.15)
CRP, mg/l		2.25 (0.90—4.55)
eGFR, ml/min/1,73m2	104.7 (19.9)	
GGTP, U/l		19.70 (12.20—37.95)
HbA1c, %		8.05 (7.15—9.90)
HDL, mmol/l	1.52 (0.34)	
LDL, mmol/l	2.90 (0.74)	
non-HDL, mmol/l	3.34 (0.92)	
NT-proBNP, pg/ml		46.05 (27.00—77.10)
TG, mg/dl		89.90 (66.43—124.88)
total cholesterol, mmol/l	4.87 (0.98)	
TSH, µIU/ml		1.57 (0.83—2.20)
uric acid, µmol/l		255.65 (200.90—325.20)
*Fatty liver disease*		
FLI		15.17 (6.84—43.07)
*Echocardiography*		
E/e'		6.75 (5.40—8.00)
EF, %	65% (4%)	
IVSD, mm	13 (2)	
LA Vol, ml	45.0 (11.4)	
LA Vol index, ml/m2	24.1 (4.8)	
LV EDV, ml	79 (15)	
LV ESV, ml	28 (6)	
LV mass, g	131.6 (35.4)	
LV mass index, g/m2	71.1 (13.0)	
LVDD, mm	46 (4)	
LVSD, mm	30 (4)	
mean e', cm/s	12 (3)	
mitral A, cm/s	64 (17)	
mitral DT, ms		230 (200 – 245)
mitral E, cm/s	81 (20)	
mitral E/A		1.25 (1.04—1.67)
RV TAM, mm	26 (3)	
RVDD, mm	25 (3)	
RWT		0.37 (0.34—0.4)
TDI bas lat a', cm/s	8 (3)	
TDI bas lat e', cm/s		14 (11 -16)
TDI bas lat s', cm/s		10 (9—12)
TDI bas sept a', cm/s		8 (7—10)
TDI bas sept e', cm/s	11 (3)	
TDI bas sept s', cm/s		8.5 (8—9)
Vp, cm/s	50 (6)	
Results are presented as mean and standard deviation (SD) for continuous variables with normal distribution and median and interquartile range (IQR) for continuous variables without normal distribution ACR albumin/creatinine ratio, mg/g; ALT alanine aminotransferase, U/l; AST aspartate aminotransferase, U/l; BMI body mass index, kg/m2; CRP C-reactive protein, mg/l; DDI daily dose of insulin, U; EF ejection fraction, %; eGFR estimated glomerular filtration rate, ml/min/1.73 m2; FLI fatty liver index; GGTP gamma-glutamyltransferase, U/l; HbA1c glycated hemoglobin, %; HDL high-density lipoprotein, mmol/l; IVSD intraventricular septum thickness at end-diastole, mm; LA Vol index left atrium volume index, ml/m2; LA Vol left atrium volume, ml; LDL low-density lipoprotein, mmol/l; LV EDV left ventricle end-diastolic volume, ml; LV ESV left ventricle end-systolic volume, ml; LV mass left ventricle mass, g; LV mass index left ventricle mass index, g/m2; LVDD left ventricular diameters at diastole, mm; LVSD left ventricular diameters at systole, mm; mitral A late diastolic mitral inflow velocity, cm/s; mitral DT deceleration time of mitral E velocity, ms; mitral E early diastolic mitral inflow velocity, cm/s; non-HDL non-high-density lipoprotein, mmol/l; NT-proBNP N-terminal prohormone of brain natriuretic peptide, pg/ml; RV TAM tricuspid annular motion, mm; RVDD right ventricular diameters at diastole, mm; RWT relative wall thickness; TDI bas lat a’ lateral mitral annulus peak late diastolic velocity, cm/s; TDI bas lat e’ lateral mitral annulus early diastolic velocity, cm/s; TDI bas sept a’ septal mitral annulus peak late diastolic velocity, cm/s, TDI bas sept e’ septal mitral annulus early diastolic velocity, cm/s; TDI bas sept s’ septal mitral annulus peak systolic velocity, cm/s; TDI lat sept s’ lateral mitral annulus peak systolic velocity, cm/s; TG triglycerides, mg/dl; TSH thyroid-stimulating hormone, µIU/ml; Vp flow velocity propagation at mitral annulus, cm/s		

There were twenty-five patients with RWT exceeding 0.42 and 30 patients with RWT of 0.42 or less. The comparison of groups with RWT > 0.42 and RWT ≤ 0.42 is presented in Table 2. The group with RWT > 0.42 differed significantly from the group without increased relative wall thickness in terms of greater FLI (40.97 vs. 13.82, *p* < 0.001), BMI (27.3 vs. 22.5 kg/m2, *p *< 0.001), and daily dose of insulin (45 vs. 40 U, *p* = 0.01), but not daily dose of insulin per kilogram body weight. Moreover, patients with increased RWT had higher concentrations of ALT (22.30 vs. 18.75 U/l, *p* = 0.005) and GGTP (34.60 vs. 17.15 U/l, *p* < 0.001) and lower AST levels (21.20 vs. 21.65 U/l, *p* = 0.04). The groups did not differ in HbA1c, NT-proBNP, HDL, LDL, non-HDL, adiponectin, APOC3, and uric acid concentrations. Additionally, the group with RWT > 0.42 had significantly higher LV mass index (85.6 vs. 68.2 g/m^2^, *p* < 0.001), TDI bas lat s’ (10 vs. 8 cm/s, *p* = 0.02) as well as lower mitral E/A (1.28 vs. 1.37, *p* = 0.04).

**Table 2:** The comparison of groups with RWT > 0.42 and RWT ≤ 0.42

Parameter	RWT > 0.42 (n = 25)	RWT ≤ 0.42 (n = 30)	*p* value
*Baseline characteristics*			
age, years (mean, SD)	39.0 (10,18)	36.3 (9.15)	0.4947
BMI, kg/m^2^ (median, IQR)	27.3 (25.3 – 28.4)	22.5 (21.2 – 27.1)	**0.00007**
body weight, kg (median, IQR)	82.5 (68.0 – 100.0)	64.0 (57.0 – 78.5)	**0.00001**
DDI per body weight kg, U/kg (median, IQR)	0.48 (0.44—0.83)	0.64 (0.5—0.82)	0.6195
DDI, U (median, IQR)	45 (32—50)	40 (35—60)	**0.0098**
diabetes duration, years (mean, SD)	20.9 (8.7)	20.9 (10.4)	0.9838
height, cm (mean, SD)	169.9 (16.1)	170.1 (9.2)	0.9636
waist circumference, cm (median, IQR)	92.0 (80.0—110.0)	80.0 (72.0 – 89.0)	**0.00003**
*Biochemistry*			
ACR, mg/g (median, IQR)	6.9 (3.8 – 18.7)	3.4 (2.5 – 7.9)	0.5214
ALT, U/l (median, IQR)	22.30 (15.40 – 48.00)	18.75 (13.80 – 27.55)	**0.0050**
AST, U/l (median, IQR)	21.10 (17.10 – 46.20)	21.65 (15.35 – 26.70)	**0.0381**
CRP, mg/l (median, IQR)	2.70 (1.10 – 4.70)	2.25 (0.90 – 5.05)	0.1025
eGFR, ml/min/1,73m^2^ (mean, SD)	93.66 (23.82)	107.29 (19.48)	0.1094
GGTP, U/l (median, IQR)	34.60 (19.90 – 126.00)	17.15 (12.20 – 30.45)	**0.0001**
HbA1c, % (median, IQR)	8.0 (7.2 – 8.8)	8.2 (7.4 – 10.1)	0.2747
HDL, mmol/l (mean, SD)	1.67 (0.35)	1.49 (0.32)	0.1284
LDL, mmol/l (mean, SD)	3.37 (0.49)	2.83 (0.76)	0.0804
non-HDL, mmol/l (mean, SD)	3.93 (0.66)	3.27 (0.94)	0.0828
NT-proBNP, pg/ml (median, IQR)	28.50 (12.80 – 76.60)	46.90 (32.30 – 105.30)	0.1462
TG, mmol/l (median, IQR)	1.39 (1.08 – 1.76)	1.02 (0.71 – 1.31)	**0.0004**
total cholesterol, mmol/l (mean, SD)	5.6 (0.91)	4.73 (0.98)	**0.0344**
TSH, µIU/ml (median, IQR)	1.61 (1.08 – 1.76)	1.30 (0.78 – 2.19)	0.5539
uric acid, umol/l (median, IQR)	251.0 (224.1 – 331.0)	258.6 (198.2 – 311.9)	0.1409
FLI	40.97 (34.31 – 83.68)	13.82 (6.32 – 40.63)	**0.0000**
*Echocardiography*			
E/e' (median, IQR)	8.0 (6.7—9.0)	6.2 (5.2 – 7.7)	0.0672
EF, % (mean, SD)	66% (6%)	65% (4%)	0.7244
IVSD, mm (mean, SD)	16 (0.5)	13 (2)	**0.0003**
LA Vol index, ml/m2 (mean, SD)	23 (3)	24 (5)	0.6738
LA Vol, ml (mean, SD)	47 (8)	45 (12)	0.5640
LV EDV, ml (mean, SD)	84 (12)	77 (16)	0.3162
LV ESV, ml (mean, SD)	30 (5)	27 (7)	0.2603
LV mass index, g/m2 (mean, SD)	85.6 (10.7)	68.2 (11.5)	**0.0007**
LV mass, g (mean, SD)	166.7 (20.2)	124.5 (33.6)	**0.0027**
LVDD, mm (mean, SD)	47 (3)	46 (5)	0.8063
LVSD, mm	29 (4)	30 (4)	0.7526
mean e', cm/s (mean, SD)	11.5 (3.7)	12.6 (2.9)	0.3894
mitral A, cm/s (mean, SD)	67 (16)	63 (17)	0.5967
mitral DT, ms (median, IQR)	239 (215—262)	227.5 (200—245)	0.5423
mitral E, cm/s (mean, SD)	83 (29)	81 (18)	0.7975
mitral E/A (mean, SD)	1.28 (0.52)	1.37 (0.43)	**0.0373**
RV TAM, mm (mean, SD)	25 (3)	27 (3)	0.1137
RVDD, mm (mean, SD)	26 (3)	25 (3)	0.3719
TDI bas lat a', cm/s (mean, SD)	8 (4)	8 (2)	0.8773
TDI bas lat e', cm/s (median, IQR)	13 (8 – 15)	14 (11 – 16)	0.0912
TDI bas lat s', cm/s (median, IQR)	10 (9—13)	8 (7—12)	**0.0186**
TDI bas sept a', cm/s (median, IQR)	9 (8 – 10)	8 (7 – 10)	0.0509
TDI bas sept e', cm/s (mean, SD)	11 (5)	11 (3)	**0.0267**
TDI bas sept s', cm/s (median, IQR)	8 (7 – 9)	9 (8—9)	0.0512
Vp, cm/s (mean, SD)	51 (8)	50 (6)	0.5709
Results are presented as mean and standard deviation (SD) for continuous variables with normal distribution and median and interquartile range (IQR) for continuous variables without normal distribution. Values in bold type are statistically significant at *p* < 0.05 *ACR* albumin/creatinine ratio, mg/g; *ALT* alanine aminotransferase, U/l; *AST* aspartate aminotransferase, U/l; *BMI* body mass index, kg/m2; *CRP* C-reactive protein, mg/l*;* *DDI* daily dose of insulin, U; *EF* ejection fraction, %*;* *eGFR* estimated glomerular filtration rate, ml/min/1.73 m2*;* *FLI* fatty liver index*;* *GGTP* gamma-glutamyltransferase, U/l; *HbA1c* glycated hemoglobin, %; *HDL* high-density lipoprotein, mmol/l*;* *IVSD* intraventricular septum thickness at end-diastole, mm*;* *LA Vol index* left atrium volume index, ml/m2*;* *LA Vol* left atrium volume, ml*;* *LDL* low-density lipoprotein, mmol/l*;* *LN APOC3* natural logarithm of apolipoprotein C3; *LV EDV* left ventricle end-diastolic volume, ml*;* *LV ESV* left ventricle end-systolic volume, ml*;* *LV mass* left ventricle mass, g*;* *LV mass index* left ventricle mass index, g/m2*;* *LVDD* left ventricular diameters at diastole, mm*;* *LVSD* left ventricular diameters at systole, mm*;* *mitral A* late diastolic mitral inflow velocity, cm/s*;* *mitral DT* deceleration time of mitral E velocity, ms*;* *mitral E e*arly diastolic mitral inflow velocity, cm/s*;* *non-HDL* non-high-density lipoprotein, mmol/l*;* *NT-proBNP* N-terminal prohormone of brain natriuretic peptide, pg/ml*;* *RV TAM* tricuspid annular motion, mm*;* *RVDD* right ventricular diameters at diastole, mm*;* *TDI bas lat a’* lateral mitral annulus peak late diastolic velocity, cm/s; *TDI bas lat e’* lateral mitral annulus early diastolic velocity, cm/s*;* *TDI bas sept a’* septal mitral annulus peak late diastolic velocity, cm/s*,* *TDI bas sept e’* septal mitral annulus early diastolic velocity, cm/s*;* *TDI bas sept s’* septal mitral annulus peak systolic velocity, cm/s*;* *TDI lat sept s’* lateral mitral annulus peak systolic velocity, cm/s*;* *TG* triglycerides, mg/dl*;* *TSH t*hyroid-stimulating hormone, µIU/ml*;* *Vp* flow velocity propagation at mitral annulus, cm/s			

*All statistically significant parameters from Student’s t-test and Mann–Whitney U test were then analysed for possible interactions. Using correlation analysis, we found a strong correlation between FLI and BMI (Spearman’s R* = *0.6876, p* = *0.0000) as well as between FLI and TG (Spearman’s R* = *0.6921, p* = *0.0000). There were no other strong correlations. Afterwards, we performed interaction analysis and found no statistically significant interactions between the remaining variables. All of them were then used in univariable logistic regression models that* revealed a positive correlation between FLI levels (OR 1.028 per unit, *p* = 0.05) as well as total cholesterol (OR 2.386 per 1 mmol/l, *p* = 0.05) and RWT. In multivariable analysis using all of the parameters none of them reached statistical significance. In the model using stepwise backward elimination, only FLI remained a statistically significant predictor. The regression models are summarized in Table 3*.*

**Table 3:** Univariable and multivariable regression models for the RWT

	*RWT*								
	*Univariable analysis*			*Multivariable analysis* *Parameters: AST, ALT, GGTP, FLI, total cholesterol, TG*			*Multivariable analysis* ▪ *Parameters: AST, ALT, GGTP, FLI, total cholesterol, TG* ▪ *Stepwise backward elimination*		
	*OR*	*SI for OR*	*p*	*OR*	*SI for OR*	*p*	*OR*	*SI for OR*	*p*
*AST, U/l*	*1.056*	*0.995 – 1.121*	*0.0702*	*1.093*	*0.954 – 1.256*	*0.199*			
*ALT, U/l*	*1.042*	*0.982 – 1.105*	*0.1714*	*0.954*	*0.832 – 1.094*	*0.501*			
*GGTP, U/l*	*1.008*	*0.996 – 1.020*	*0.1865*	*0.993*	*0.972 – 1.014*	*0.512*			
*FLI*	***1.028***	***1.001 – 1.056***	***0.045***	*1.026*	*0.991 – 1.062*	*0.145*	***1.028***	***1.001 – 1.056***	***0.045***
*total cholesterol, mmol/l*	***2.386***	***1.011 – 5.63***	***0.0472***	*1.612*	*0.581 – 4.472*	*0.359*			
Values in bold type are statistically significant at *p* < 0.05 *ALT* alanine aminotransferase, U/l; *AST* aspartate aminotransferase, U/l; *FLI* fatty liver index*;* *GGTP* gamma-glutamyltransferase, U/l;									

The suggested cutoff point for FLI predicting the RWT > 0.42 using the Youden index was 34.31 (sensitivity 0.857, specificity 0.686) which offered AUC 0.784. In Persons’s Chi2 test, FLI > 34.31 significantly correlated with RWT > 0.42 with *p* = 0.01 and OR 12.55, *p* = 0.03.

## Discussion

This study provides a few findings concerning LV remodeling in patients with T1D and the FLI potential in identifying patients with T1D at its higher risk.

In our study, about 45% of participants presented with concentric remodeling recognized as RWT > 0.42 and correct LV mass index, which is more than in other previously studied populations. Namely, in a study describing 694 patients with essential hypertension, this ratio was less than 40% [23]. The prevalence of concentric remodeling in T1D patients remains vague, and the known estimates vary from 12 to 67% [1, 5–7, 24].

A hallmark of diabetic cardiomyopathy, HFpEF development is characterized by a progressive evolution from restricted, concentric left ventricle remodeling through HF with reduced EF (HFrEF) to eccentric hypertrophy[14, 25–27]. Multiple studies suggest that concentric remodeling is associated with an increased rate of cardiovascular events, stroke, morbidity, and mortality[10, 12]. Regardless of concomitant LV systolic dysfunction, mounting evidence from epidemiological studies implies that patients with diastolic dysfunction impart poor prognostic implications, with threefold increased mortality when compared to non-diabetic individuals[25].There are no screening protocols for identifying T1D patients at risk of diabetic cardiomyopathy, and none of the previously proposed methods, e.g., B-type natriuretic peptide, were considered satisfactorily sensitive and specific(6).

In our study, 20% of participants presented with a high risk of MASLD, which matches the available literature data. MAFLD’s estimated prevalence among adults equals to 15–30% in the general population, while it may reach 60–70% among patients with T2D[28, 29]. At the same time, as reported, up to 40% of T1D patients present with MASLD features[30]. We did not observe this tendency in our population though, as the percentage of participants with a high risk of MASLD resembled the general population more.

There are multiple common denominators between T1D, MASLD, and diabetic cardiomyopathy. As previously described, patients with newly diagnosed T1D already exhibit increased insulin resistance independently of their BMI, physical activity, and fat distribution [31]. The exact underlying process of this phenomenon remains undetermined. Nevertheless, some researchers suggest that T1D may be accompanied by reduced hepatic mitochondrial activity either due to loss of portal/peripheral insulin gradient or specific variations in genes controlling oxidative metabolism such as PGC1α [31–34]. Other possible explanations include that insulin resistance may stem from relative hepatic insulin deficiency accompanying peripheral hyperinsulinemia or diminishing glucagon secretion suppression [31–34]. In T1D, increased hepatic insulin resistance may consequently expedite the rate of intrahepatic fat deposition [30].

Furthermore, MASLD correlate with impaired vasomotor actions, accelerated atherosclerosis, and coronary artery calcification and may additionally fuel insulin resistance [35]. At the same time, heart dysfunction in T1D is considered a result of interactions between metabolic disturbances, including hyperglycemia, insulin deficiency, and insulin resistance and mounting advanced glycation end products, endothelial dysfunction, and cellular abnormalities resulting in increased oxidative stress, cardiomyocyte dysfunction, cardiac inflammation, and remodeling [1, 27, 36]. Mentioned pathological processes potentially form a feedback loop fueling each other.

In previously described populations, every additional 1% of HbA1c correlated with the 30% HF risk increase, independently of other cardiovascular risk factors [37]. In our study group, we did not notice significant correlations between metabolic control parameters or diabetes duration and RWT. This finding may support a hypothesis of different mechanisms leading to concentric remodeling among patients with T1D [37]. An expanding body of evidence proves that diabetic cardiomyopathy may be present early in the course of the disease, even in pediatric populations, despite strict glycemic control [36]. Studies depicting the association between diabetes metabolic control and RWT concern primarily patients with T2DM, and we hypothesize that this particular difference may be due to different pathophysiological mechanisms underlying different diabetes types [38].

In our analysis, T1D patients with concentric remodeling had significantly higher FLI. In further analysis, every 1 unit increase in FLI over 34.31 correlated with a 2.8% increase in concentric remodeling odds ratio. Interestingly, using FLI > 26.38 as a cut-off point, which lands in the MASLD low-risk category, led to similar outcomes. In a large population study, a significant association between FLI with HF and mortality was described, with hazard ratio increased by 33% and 81%, respectively, in patients with diabetes [39]. Interestingly, these associations were still present in the non-diabetic cohort, but their magnitude was distinctly reduced [39]. Contrary to traditional conceptualization, liver steatosis was described even in normal-weight populations [29]. Another study reported the correlation between FLI and LV hypertrophy and diastolic heart function independent of other risk factors, including blood pressure and obesity [40].

Study participants differed significantly in terms of LV mass index. This finding may be explained partially by varying FLI scores. Studies of large populations showed that a high risk of MASLD correlates with LV mass index and more severe cardiac and liver fibrosis [15, 28].

In our study group, patients differed in ALT, AST, and GGTP concentrations, but their median values were still within a normal range in both groups. This observation stays in line with previously published research [41] However, patients with RWT > 0.42 had significantly higher total cholesterol and triglyceride levels despite no differences between HDL, LDL, and non-HDL concentrations. This finding adds to conflicting data on the relationship between lipids and LV remodeling. Multiple studies present significant correlations between particular lipid fractions and RWT in numerous groups [42, 43]. On the other hand, Bjelakovic et al., in a recent study, reported no significant correlations between RWT and total cholesterol, triglycerides, LDL, or HDL [44]. We hypothesize that the discrepancy between our and reported results may be due to the very characteristic of lipid profile alterations in T1D. Patients with T1D, even with satisfactory glycemic control, present a profile of lipid abnormalities with a detrimental effect on CV risk and a possible potential to promote left ventricular remodeling through increased rates of lipid accumulation in myocardial cells and a surge in insulin resistance and oxidative stress, for instance, triglyceride enrichment of LDL or increased percentage of serum amyloid A in HDL [45]. In our opinion, this issue is worth further exploration.

In our opinion, recognizing concentric remodeling in patients with T1D may be useful in determining the risk of HF and facilitate the identification of patients needing early lifestyle interventions that may mitigate this risk, as the concentric remodeling is seemingly a reversible, modifiable factor [46, 47]. Moreover, we suppose that our findings further strengthen the universal need to monitor patients’ weight closely and provide them with appropriate tools and support when they surpass the overweight or obese categories. Moreover, it may serve as an encouragement to pay closer attention to patients presenting with signs of so-called double diabetes (DD; diabetes type 1 with metabolic syndrome characteristics), which considerably experiences a surge in prevalence [48].

Several limitations to this study must be acknowledged. First, it is a relatively small single-center study, designed to establish a basis for further larger research. Therefore, further longitudinal studies on larger cohorts are needed to gather more thorough data. Second, patients treated for additional disorders accounted for less than 25% of our study group, and as a result, we were not able to adequately incorporate this data into statistical analyses. As we acknowledge that all the additional medications may influence cardiac performance, we are convinced that it should be further explored in larger cohorts. Third, due to our limited organizational capacity, instead of a gold-standard liver biopsy, we used a surrogate index of MAFLD risk, albeit validated against the mentioned gold standard and offering acceptable sensitivity and specificity. Lastly, in our opinion, further longitudinal studies on larger cohorts are necessary to more extensively explore correlations of study parameters and models on LV remodeling.

## Conclusions

FLI correlates positively with relative wall thickness in patients with type 1 diabetes independently of diabetes glycemic control. The FLI > 34.3, which reflects an intermediate MAFLD risk, considerably increases the odds ratio for concentric remodeling (OR 12.55). FLI supposedly may serve as a tool supporting clinicians in identifying patients at higher risk of LV remodeling requiring closer monitoring. Nevertheless, further research is needed to support this proposition.

## Data Availability

The datasets used and/or analysed during the current study are available from the corresponding author on reasonable request.
